# Efficacy of Gut Microbiome-Targeted Therapies in Modulating Systemic Inflammation and Low-Grade Chronic Inflammatory States in Adults With Metabolic Disorders: A Systematic Review

**DOI:** 10.7759/cureus.92881

**Published:** 2025-09-21

**Authors:** Abdulkreem Al-Juhani, Mahmoud S Desoky, Amaal A Almuhaimid, Memona Zaheer, Hoor F Alhaqbani, Elaf A Abalkhail, Sama A Alanazi, Ruof S Alzahrani, Mamoun Alrefaai, Rodan Desoky

**Affiliations:** 1 Forensic Medicine Department, Forensic Medicine Center, Jeddah, SAU; 2 Department of Surgery, King Abdulaziz University Faculty of Medicine, Jeddah, SAU; 3 Internal Medicine, Gastroenterology, Sultan Bin Abdulaziz Humanitarian City, Riyadh, SAU; 4 College of Medicine, Princess Nourah bint Abdulrahman University, Riyadh, SAU; 5 Medicine, Batterjee Medical College, Jeddah, SAU; 6 Medicine and Surgery, Princess Nourah Bint Abdulrahman University, Riyadh, SAU; 7 Medical School, AlMaarefa University, Riyadh, SAU; 8 College of Medicine, Internal Medicine, AlMaarefa University, Riyadh, SAU; 9 Medicine, Alfaisal University College of Medicine, Riyadh, SAU

**Keywords:** fecal microbiota transplantation (fmt), gut microbiome, inflammatory markers, low-grade chronic inflammation, metabolic syndrome, obesity, prebiotics, probiotics, synbiotics, type 2

## Abstract

Gut microbiome-targeted therapies have shown promise in promoting the outcomes of metabolic inflammation-related disease management. This review aims to assess the effectiveness of microbiome-targeted interventions and pinpoint the most promising therapies for clinical implementation. Following the PRISMA 2020 standards, we searched four main databases: PubMed, EMBASE, Scopus, and the Cochrane Library. We integrated medical subject headings (MeSH) and free-text keywords search pertinent to gut microbiome-targeted interventions, along with related outcomes such as inflammation and insulin resistance. English studies were conducted on primary adults with a metabolic disease diagnosis or deemed a high risk, and were mandated to report at least one outcome pertinent to metabolic health or systemic inflammation. To assess the risk bias, data were extracted, and the Cochrane Risk of Bias (RoB) 2.0 tool was employed. Fifteen studies fulfilled the inclusion criteria. A narrative synthesis was conducted. We found that probiotics significantly enhanced insulin resistance (HOMA-IR), reduced circulating endotoxin levels, decreased visceral fat, BMI, and fat mass, and increased beneficial taxa with obesity-associated bacteria reduction. However, inconsistent outcomes were shown for lipid parameters. Prebiotic therapies showed significant decreases in fasting glucose in overweight people, and insulin levels and HOMA-IR in patients with metabolic syndrome, enhanced anti-inflammatory effects (31% C-reactive protein (CRP) reduction, decreased interleukin (IL)-6, tumor necrosis factor-alpha (TNF)-α, and lipopolysaccharide (LPS) levels), and promoted butyrate-producing bacteria. Synbiotic interventions showed complementary benefits for glucose metabolism and body composition. Fecal microbiota transplantation (FMT) studies indicated improved insulin sensitivity and donor microbiota engraftment in responders. Fiber-rich diet trials markedly improved HbA1c levels in diabetic and prediabetic individuals. In conclusion, prebiotics demonstrated the most consistent metabolic and anti-inflammatory benefits across multiple parameters. Probiotics showed targeted effects on insulin resistance and body composition but inconsistent lipid outcomes. FMT and synbiotics require further investigation to establish clinical efficacy. This evidence supports prebiotics as a priority intervention for metabolic disease management through microbiome modulation.

## Introduction and background

Metabolic illnesses, including obesity, metabolic syndrome, type 2 diabetes (T2D), and non-alcoholic fatty liver disease (NAFLD), represent significant global health issues. A prevalent characteristic among these disorders is persistent low-grade systemic inflammation, which is instrumental in the onset of insulin resistance, hepatic steatosis, and vascular dysfunction [1]. In recent years, the gut microbiome has become a crucial regulator of host metabolism and immunological function, with increasing evidence associating microbial dysbiosis with the etiology of metabolic diseases [2,3]. Individuals with metabolic disorders frequently exhibit alterations in gut microbial composition, characterized by a pro-inflammatory profile, diminished microbial diversity, a decline in beneficial taxa (such as* Bifidobacterium* and *Faecalibacterium*), and an increased prevalence of opportunistic pathogens. Dysbiosis may enhance intestinal permeability, facilitating the translocation of microbial components like lipopolysaccharide (LPS) into the bloodstream, termed metabolic endotoxemia, which initiates systemic inflammation through Toll-like receptor activation [3]. 

Furthermore, a dysbiotic microbiota may diminish the synthesis of advantageous metabolites such as short-chain fatty acids (SCFAs), which typically possess anti-inflammatory and insulin-sensitizing properties [4]. The molecular discoveries have generated heightened interest in gut microbiome-targeted treatments as potential means to control metabolic inflammation and enhance cardiometabolic outcomes [5]. Numerous human clinical trials have investigated interventions including probiotics (live beneficial bacteria), prebiotics (fermentable fibers that enhance beneficial microbes), synbiotics (combinations of both), fecal microbiota transplantation (FMT), and dietary strategies (e.g., high-fiber or fermented foods) designed to modify the gut microbiota [6,7]. Probiotics have been linked to decreased inflammatory markers, including C-reactive protein (CRP), as well as enhancements in glycemic management, lipid profile, and body composition in individuals with obesity or T2D [1,7]. Synbiotics and prebiotics have demonstrated further potential in improving gut barrier integrity and mitigating liver inflammation in NAFLD [8].

FMT has arisen as a more direct method for microbiota manipulation. A pivotal randomized controlled research revealed that lean donor FMT enhanced insulin sensitivity in obese insulin-resistant individuals; however, the effect was temporary and contingent on the donor [8]. Novel microbial possibilities, including *Akkermansia muciniphila*, have been investigated in humans, with supplementation resulting in enhanced insulin sensitivity, decreased inflammatory markers, and modest weight reduction in overweight persons [9]. Notwithstanding these promising results, the therapeutic efficacy of medicines aimed at gut microbiota remains variable. Certain randomized trials indicate substantial metabolic and anti-inflammatory advantages, but others have negligible or no impact [10]. Responses may differ based on individual microbiome composition, dietary habits, genetic variables, or other host characteristics. Meta-analyses indicate moderate yet substantial enhancements in specific metabolic parameters with probiotics and synbiotics, whereas evidence for newer approaches such as FMT is still limited [7,11]. An umbrella analysis of systematic reviews indicated that whereas microbiome therapies frequently affect metabolic indicators, the overall robustness and consistency of the evidence remains under development [12]. 

A thorough synthesis of the data is necessary due to the range of interventions and the differing outcomes reported in studies. This systematic review seeks to assess the effectiveness of gut microbiome-targeted interventions, such as probiotics, prebiotics, synbiotics, dietary modifications, and FMT, in diminishing systemic inflammation and enhancing metabolic health in individuals with obesity, T2D, metabolic syndrome, or NAFLD. By synthesizing findings from various therapy modalities and inflammatory/metabolic outcomes, we seek to evaluate the existing evidence strength and pinpoint the most promising options for clinical implementation.

## Review

Methodology 

This systematic review was executed in accordance with the Preferred Reporting Items for Systematic Reviews and Meta-Analyses (PRISMA) 2020 principles to guarantee transparency and methodological precision. A thorough search approach was implemented across four primary databases, i.e., PubMed, EMBASE, Scopus, and the Cochrane Library, to locate pertinent literature published until August 2025. 

The search integrated medical subject headings (MeSH) and free-text keywords pertinent to gut microbiome-targeted interventions (e.g., “probiotics”, “prebiotics”, “synbiotics”, “fecal microbiota transplantation,” “dietary fiber”) and metabolic disorders (e.g., “obesity”, “type 2 diabetes”, “metabolic syndrome”, “NAFLD”), along with related outcomes such as inflammation and insulin resistance. 

The review concentrated solely on primary human research published in English. Studies were eligible for inclusion if they involved adults (aged ≥18 years) diagnosed with a metabolic illness or deemed at high risk and evaluated therapies designed to modulate the gut microbiota in comparison to placebo, conventional care, or an alternative microbiome intervention. Eligible studies were mandated to report at least one outcome pertinent to metabolic health (e.g., BMI, fasting glucose, HbA1c, lipid profile, liver enzymes, insulin sensitivity) or systemic inflammation (e.g., CRP, IL-6), and could additionally encompass mechanistic markers such as gut microbiota composition or short-chain fatty acid concentrations. 

Studies were eliminated if they involved animal or in vitro research, secondary analyses (e.g., reviews or meta-analyses), abstracts lacking full texts, and non-English publications or were conducted in pediatric populations. Two separate reviewers performed title/abstract screening, full-text evaluation, and data extraction utilizing a standardized template, resolving discrepancies through discussion. The extracted data encompassed study characteristics, participant demographics, intervention specifics, comparator descriptions, and reported outcomes. The Cochrane Risk of Bias (RoB) 2.0 tool was employed to evaluate the RoB in randomized trials. 15 studies fulfilled the inclusion criteria. 

**Figure 1:**
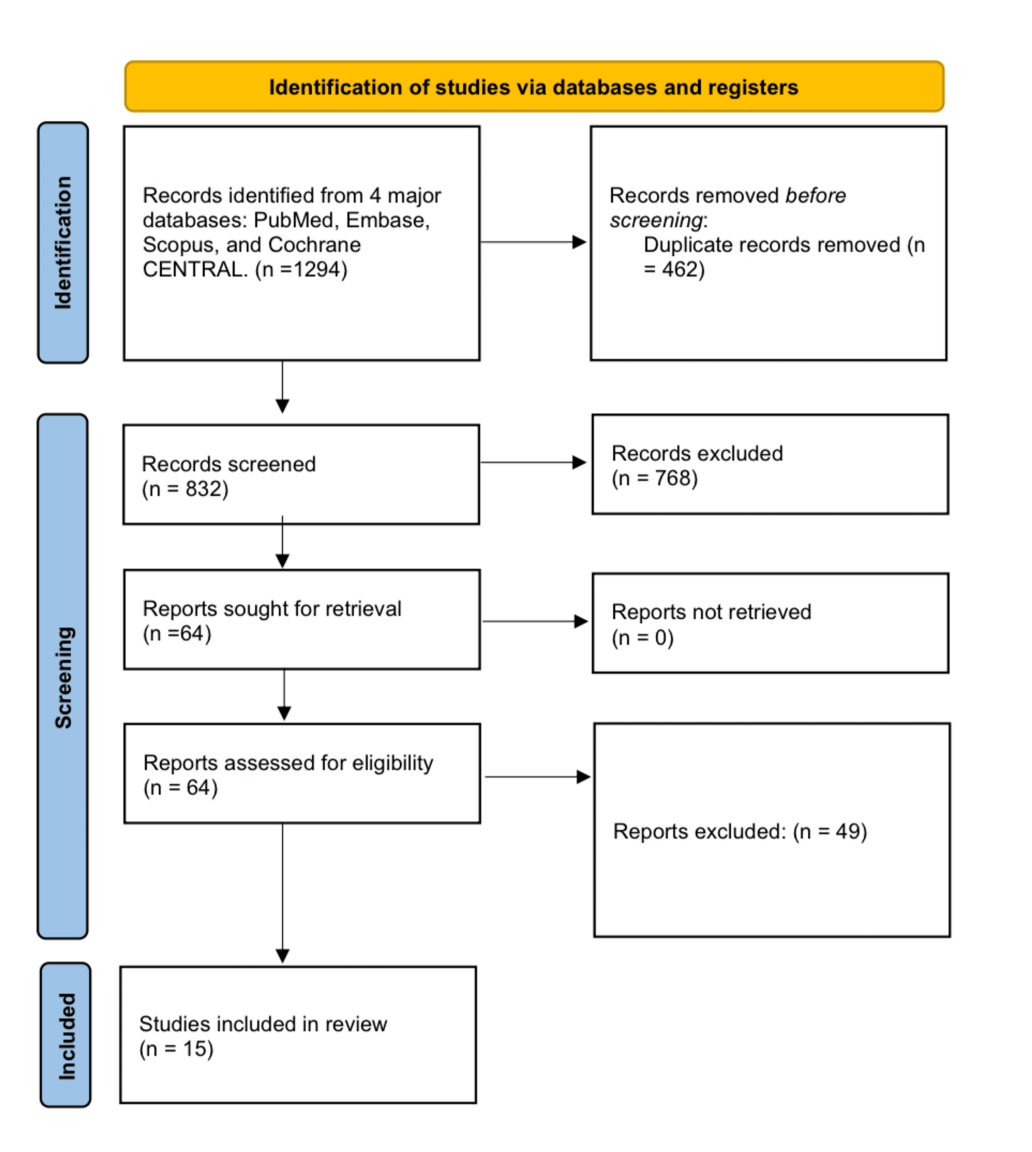
Preferred Reporting Items for Systematic Reviews and Meta-Analyses (PRISMA) flow diagram

Due to the variety in study designs, demographics, and outcome measures, a narrative synthesis approach was employed to synthesize the results, categorized by intervention type and clinical condition (Figure 1).

Risk-of-Bias Assessment 

The RoB was evaluated across seven dimensions utilizing the Cochrane RoB 2 methodology for all 15 included randomized controlled trials. The quality of the included studies ranged from mediocre to high. Eleven studies (73%) were assessed as having a low RoB across all dimensions, signifying robust methodological rigor. These trials consistently demonstrated sufficient randomization, participant blinding, assessor blinding, and comprehensive outcome data. Four studies (27%) were assessed as having a moderate RoB, mostly due to ambiguous documentation of random sequence generation, allocation concealment, or selective result reporting. No study was considered to possess a high RoB in any domain. The findings indicate that although the majority of the included trials were methodologically robust, greater transparency in randomization processes and protocol registration could elevate the quality of future research (Table 1).

**Table 1:** Risk-of-bias assessment table (RoB 2 tool)

Study	Randomization process	Allocation concealment	Blinding of participants and personnel	Blinding of outcome assessment	Incomplete outcome data	Selective reporting	Overall risk of bias
Wastyk et al., 2023 [1]	Low risk	Low risk	Low risk	Low risk	Low risk	Low risk	Low risk
Kadooka et al., 2013 [2]	Medium risk	Medium risk	Low risk	Low risk	Low risk	Medium risk	Medium risk
Peng et al., 2024 [3]	Low risk	Low risk	Low risk	Low risk	Low risk	Low risk	Low risk
Sabico et al., 2017 [4]	Low risk	Low risk	Low risk	Low risk	Low risk	Low risk	Low risk
Tsai et al., 2025 [5]	Low risk	Low risk	Low risk	Low risk	Low risk	Low risk	Low risk
Hiel et al., 2020 [6]	Medium risk	Medium risk	Medium risk	Low risk	Low risk	Low risk	Medium risk
Reimer et al., 2017 [7]	Low risk	Low risk	Low risk	Low risk	Low risk	Low risk	Low risk
Zolghadrpour et al., 2024 [8]	Low risk	Low risk	Low risk	Low risk	Low risk	Low risk	Low risk
Zhao et al., 2018 [9]	Medium risk	Medium risk	Medium risk	Low risk	Low risk	Low risk	Medium risk
Dehghan et al., 2014 [10]	Low risk	Low risk	Medium risk	Low risk	Low risk	Low risk	Low risk
Vrieze et al., 2012 [11]	Low risk	Low risk	Low risk	Low risk	Medium risk	Medium risk	Medium risk
Kootte et al., 2017 [12]	Low risk	Low risk	Low risk	Low risk	Low risk	Medium risk	Medium risk
Yu et al., 2020 [13]	Low risk	Low risk	Low risk	Low risk	Low risk	Low risk	Low risk
Ng et al., 2022 [14]	Low risk	Low risk	Low risk	Low risk	Low risk	Low risk	Low risk
Wu et al., 2025 [15]	Low risk	Low risk	Low risk	Low risk	Low risk	Low risk	Low risk

Results

Glycemic Control 

Of the 15 studies included, 10 evaluated glycemic control outcomes, including fasting blood glucose (FBG), insulin levels, or HbA1c. In probiotic trials (Table 2), one study involving newly diagnosed T2D patients demonstrated a substantial enhancement in insulin resistance (HOMA-IR) with a multi-strain probiotic compared to placebo (Table 2). However, other studies indicated no significant advantage over placebo in HbA1c or FBG levels. Prebiotic and synbiotic research (Table 3) exhibited more consistent outcomes; inulin-type prebiotics and synbiotic yogurt resulted in significant decreases in fasting glucose, insulin levels, and HOMA-IR in overweight people and patients with metabolic syndrome, respectively (Table 3). Dehghan et al. [10] observed enhanced glycemic indices and HbA1c levels with inulin supplementation in women with T2DM. Fiber-rich diets markedly improved HbA1c levels in individuals with T2DM and prediabetes, while one FMT experiment indicated increased insulin sensitivity following lean donor transfer in patients with metabolic syndrome. Conversely, some FMT trials did not demonstrate sustained glycemic advantages.

**Table 2:** Summary of probiotic intervention studies

Study (author, year)	Country	Study design	Population (N)	Intervention	Comparator	Duration	Outcomes	Key findings	Year
Wastyk et al., 2023 [1]	USA	Randomized double-blind placebo-controlled trial (1:1)	39 adults with metabolic syndrome risk factors	Daily multi-strain probiotic supplement (formulated for metabolic syndrome; ~20 billion CFU, 3 strains)	Placebo	18 weeks	Metabolic syndrome parameters (blood pressure, lipids, glucose, etc), immune markers, gut microbiome profiles	No overall improvement in metabolic syndrome markers vs placebo. However, a subset of probiotic “responders” showed significant ↓ triglycerides and ↓ diastolic BP, whereas “non-responders” had ↑ blood glucose and insulin. Responders exhibited a distinct gut microbiome and better diet, suggesting probiotic benefits depended on diet.	2023
Kadooka et al., 2013 [2]	Japan	Multi-center double-blind RCT (3-arm parallel)	210 healthy Japanese adults with high visceral fat (abdominal adiposity)	Fermented milk with Lactobacillus gasseri SBT2055 at two doses (10^7 or 10^6 CFU/g; 200 g/day)	Placebo fermented milk (no live LG2055)	12 weeks (+4-week follow-up)	Visceral fat area (CT); body weight and composition (BMI, waist/hip circumference, fat mass)	Visceral abdominal fat was significantly reduced (~−8% from baseline) in both probiotic groups by 12 weeks, with concurrent ↓ BMI, waist/hip size, and fat mass. The placebo group had no significant reductions. Benefits waned after cessation (4 weeks off), suggesting continuous intake is needed to maintain fat-loss effects.	2013
Peng et al., 2024 [3]	China	Randomized double-blind placebo-controlled trial (1:1)	213 adults with type 2 diabetes mellitus (hospital-based)	Lactobacillus-based probiotic drink (25 mL, >10^8 CFU/mL) four times daily	Placebo drink (25 mL ×4, heat-inactivated bacteria)	16 weeks	Glycemic control (HbA1c, fasting blood glucose); lipid profile; body weight; insulin resistance markers	After 16 weeks, both groups showed mild reductions in HbA1c and FBG, with no statistically significant difference between probiotic vs placebo (P>0.05). Similarly, there were no significant probiotic benefits on lipid profile or body weight. In summary, probiotic supplementation provided no added improvement in glycemic control, lipids, or weight compared to placebo.	2024
Sabico et al., 2017 [4]	Saudi Arabia	Single-center double-blind randomized trial	78 Saudi adults with newly diagnosed T2DM (medication-naïve, no comorbidities)	Multi-strain probiotic (Ecologic®Barrier, 8 strains Bifidobacterium and Lactobacillus, ~2.5×10^9 CFU/g) twice daily	Placebo sachet (inactive filler, twice daily)	12 weeks	Endotoxin (LPS); insulin resistance (HOMA-IR); anthropometrics (waist–hip ratio); metabolic profile	Probiotic did not significantly reduce endotoxin (LPS) vs placebo (p=0.15). However, the probiotic group had a marked improvement in insulin sensitivity (HOMA-IR ↓60% vs ↓12% in placebo; p=0.04) and a modest reduction in waist–hip ratio (p=0.02). No serious adverse events; probiotic was safe.	2017
Tsai et al., 2025 [5]	Taiwan	Randomized double-blind placebo-controlled trial	59 overweight adults (BMI 25–29.9, age 20–40; excess body fat)	Lactobacillus plantarum GKM3 probiotic (capsules, 1 g/day)	Placebo capsules	4 weeks	GI function (bowel movement frequency, GI symptom scores); fecal fat excretion; gut microbiota	GKM3 probiotic significantly increased bowel movement frequency and alleviated GI symptoms (reflux, nausea, abdominal pain, constipation) vs placebo. Fat absorption: higher fecal triglyceride/cholesterol indicated reduced fat absorption. Microbiome: beneficial shifts (↓ obesity-associated taxa; ↑ Akkermansia and Lactobacillus).	2025

Inflammatory and Endotoxemia Markers

Six studies assessed indicators associated with inflammation or endotoxins. In the probiotic cohort, one study indicated a 60% enhancement in insulin sensitivity, alongside a slight albeit statistically insignificant decrease in circulating endotoxin (LPS) (Table 2). Prebiotic therapies demonstrated enhanced anti-inflammatory effects: inulin supplementation decreased IL-6, TNF-α, and plasma LPS levels and led to a 31% drop in C-reactive protein (CRP), although not all alterations reached statistical significance (Table 3). Synbiotic yogurt resulted in decreased systemic inflammation, as indirectly shown by enhanced metabolic syndrome markers. No FMT trial specifically assessed inflammatory biomarkers; nevertheless, enhancements in insulin sensitivity and lipid metabolism may indicate decreased inflammation (Table 4).

**Table 3:** Prebiotic fiber intervention studies

Study (author, year)	Country	Study design	Population (N)	Intervention	Comparator	Duration	Outcomes	Key findings
Hiel et al., 2020 [6]	Belgium	Randomized, single-blind, multicenter placebo-controlled trial	Obese adults (N=150)	Native inulin (16 g/day) + dietary advice (inulin-rich veggies) + caloric restriction	Maltodextrin placebo (16 g/day) + dietary advice (inulin-poor veggies) + caloric restriction	3 months	Weight, blood pressure, liver enzymes, insulin, gut microbiota	The insulin group had greater weight loss and additional drops in diastolic BP, AST, and fasting insulin vs placebo. Inulin also modulated gut microbiota (↑ Bifidobacterium, ↓ Desulfovibrio), changes associated with improved anthropometry. Metformin use blunted many microbiota/metabolic benefits.
Reimer et al., 2017 [7]	Canada	Randomized, double-blind, placebo-controlled trial (4-arm)	Overweight/obese adults (N=125)	Inulin-type fructans (ITF) snack bars (± added whey protein)	Placebo snack bars (isocaloric, no added fiber/protein)	12 weeks	Appetite ratings, body composition, gut microbiota	All intervention groups (ITF, Whey, ITF+Whey) showed reduced hunger/appetite vs control. Whey-only reduced body fat vs control. Prebiotic ITF (alone/with whey) altered gut microbiota (↑ Bifidobacterium); appetite improved with all treatments, but only fructan arms induced microbiota changes.
Zolghadrpour et al., 2024 [7]	Iran	Randomized, placebo-controlled trial	Adults with metabolic syndrome (N=41)	Synbiotic yogurt (300 g/day; L. plantarum, L. pentosus, C. marcusianus + prebiotic herbs)	Regular yogurt (300 g/day) without added synbiotics	12 weeks	Metabolic syndrome components (glycemia, insulin resistance, BP, anthropometrics)	Synbiotic yogurt significantly improved insulin resistance markers—fasting glucose, fasting insulin, and HOMA-IR decreased. Greater reductions in waist-to-hip ratio and systolic BP vs plain yogurt. Suggests daily synbiotic yogurt favorably modulates metabolic parameters via anti-inflammatory/microbiota-mediated effects.
Zhao et al., 2018 [9]	China	Randomized trial (open-label, two-arm)	Patients with type 2 diabetes (N≈43; on acarbose)	High-fiber diet (diverse fermentable fibers) + standard care (including acarbose)	Standard diet recommendations + standard care (acarbose)	12 weeks	Glycemic control (HbA1c, fasting glucose), weight, lipid profile, gut microbiota	A high-fiber diet achieved greater improvements in glycemic control and body weight than controls. HbA1c fell more, fasting glucose declined faster, and weight loss was greater vs. the standard diet. The fiber selectively promoted ~15 of 141 SCFA-producing strains, increasing butyrate/acetate and improving GLP-1–mediated glucose regulation, insulin sensitivity, and lipid metabolism.
Dehghan et al., 2014 [10]	Iran	Randomized, placebo-controlled trial	Overweight women with type 2 diabetes (N=52)	Oligofructose-enriched inulin (10 g/day)	Maltodextrin (10 g/day)	8 weeks	Glycemic indices (FPG, HbA1c), inflammatory cytokines (IL-6, TNF-α, etc.), endotoxemia (plasma LPS)	Inulin significantly decreased FPG (~−9.5%), HbA1c (~−8.4%), IL-6 (~−8%), TNF-α (~−20%), and plasma LPS (~−22%) vs baseline and vs maltodextrin (all P<0.05). Other markers (IFN-γ, IL-10, hsCRP) were favorable (e.g., CRP ↓31%, IL-10 ↑11%) but not significant vs placebo.

Lipid Profile

Lipid parameter alterations were evaluated in eight trials. Probiotic therapies yielded inconsistent outcomes; one study noted decreased triglycerides in a specific group of probiotic responders, while others reported no meaningful impact on lipid profiles (Table 2). Prebiotic and synbiotic trials exhibited modest enhancements in lipid metabolism, namely, reductions in LDL and triglycerides in the fiber and synbiotic groups (Table 3). Studies combining FMT with lifestyle modifications and high-fiber diet interventions demonstrated enhancements in LDL levels (Table 4).

**Table 4:** Fecal microbiota transplantation (FMT) and diet-based microbiome interventions

Study (Author, Year)	Country	Design	Population (N)	Intervention	Comparator	Duration	Outcomes	Key Findings (concise)	Year
Vrieze et al., 2012 [11]	Netherlands	RCT	Males with MetS (n=18)	Single lean donor FMT	Autologous FMT	6 weeks	Insulin sensitivity; microbiota shifts	Lean-donor FMT improved insulin sensitivity and increased butyrate producers vs control.	2012
Kootte et al., 2017 [12]	Netherlands	Double-blind RCT	Males with MetS (n=38)	Lean-donor FMT	Autologous FMT	18 weeks	Insulin sensitivity; gut taxa	Transient metabolic benefit at 6 weeks; baseline microbiota predicted FMT responders.	2017
Yu et al., 2020 [13]	USA	Placebo-controlled RCT	Obese, insulin-resistant adults (n=24)	Weekly oral FMT capsules (6 weeks)	Placebo capsules	12 weeks	Engraftment; insulin sensitivity	Safe donor engraftment, but no improvement in insulin resistance or glycemic markers vs placebo.	2020
Ng et al., 2022 [14]	Hong Kong	3-arm RCT	Obese adults with T2DM (n=61)	FMT + lifestyle intervention	FMT-only; sham + lifestyle	24 weeks	LDL, liver stiffness, gut microbiota	FMT + lifestyle improved LDL and microbiota engraftment better than FMT alone.	2022
Wu et al., 2025 [15]	Singapore	Single-blind RCT	Prediabetic Chinese adults (n=127)	Calorie-restricted, legume-rich intervention diet	Isocaloric standard diet	16 weeks	HbA1c, LDL, SCFA, bile acids, microbiota	Greater reductions in HbA1c and LDL; mediated via shifts in fiber degraders and microbial metabolites.	2025

Anthropometric Measures

Eleven studies evaluated outcomes including body weight, BMI, and waist-to-hip ratio. Two probiotic trials demonstrated substantial decreases in visceral fat, BMI, and fat mass with the administration of probiotic milk or capsules; however, one study observed that the effects faded following the cessation of supplementation (Table 2). Prebiotic research generally demonstrated weight loss and reduced central adiposity, particularly when accompanied by dietary guidance or caloric restriction (Table 3). Synbiotic yogurt and inulin-type fructans were correlated with enhanced body composition. High-fiber and legume-rich diets led to substantial weight loss and decreased waist circumference in prediabetic and obese individuals among diet-based therapies (Table 4).

Gut Microbiota Composition and SCFA Production

Microbiome changes were documented in nine studies. Probiotics and prebiotics often increased beneficial taxa (e.g., *Bifidobacterium*, *Akkermansia*) and reduced obesity-associated bacteria (Tables 2, 3). In one probiotic trial, responders had distinct baseline microbiota, suggesting personalized effects. Prebiotics selectively enhanced short-chain fatty acid (SCFA) producers, contributing to increased butyrate and acetate levels. FMT studies confirmed engraftment of donor microbiota and increased abundance of butyrate-producing bacteria in responders, although not all studies observed clinical benefit (Table 4). Diet-based interventions led to enrichment of fiber-degrading and SCFA-producing taxa, supporting their mechanistic role in metabolic improvement.

Discussion 

Golden discoveries in randomized trials involving individuals with obesity, metabolic syndrome, T2DM, and NAFLD produced modest enhancements in domain-specific microbiome-targeted treatments, instead of comprehensive metabolic reversal [13-15]. In our dataset, prebiotics and synbiotics consistently enhanced insulin resistance and central adiposity, whereas the effects of probiotics on glycaemia and lipids were variable and frequently dependent on the host and diet. The advantages of FMT seemed to be contingent on context and transient until integrated with lifestyle modifications. 

These trends correspond with extensive data connecting microbiome composition, metabolic inflammation, and short-chain fatty acid biology to insulin sensitivity and obesity [16-19]. Context accompanied by preceding evidence. Human and multi-omic research indicate that microbiome diversity, functional capacity, and metabolite production (particularly short-chain fatty acids) are associated with insulin sensitivity and metabolic risk [20-24]. Targeted fiber/SCFA interventions can diminish hunger and enhance adiposity and glycemic control in regulated environments [19-21, 25]. Expert consensus currently characterizes prebiotics as substrates selectively metabolized by host microorganisms that provide a health advantage [26]. 

Simultaneously, empirical probiotics encounter individualized colonization resistance and inconsistent efficacy, elucidating the phenomenon of "responders" and "non-responders" observed clinically [22,27]. Novel microbes like *Akkermansia muciniphila *exhibit preliminary indications of insulin sensitivity and inflammation in humans, corroborated by mechanistic studies [28-31]. Mechanistic feasibility. Enhancements in glycaemia, adiposity, and inflammation noted in fibre-centric interventions likely indicate effects mediated by SCFAs (GLP-1 signalling, hepatic and adipose reprogramming), diminished endotoxaemia, and restoration of barriers, in accordance with causal and integrative data [19-21,23,32-34]. Fundamental research links dysbiosis and diminished microbial diversity to negative metabolic phenotypes and host metabolome profiles [13-17,33,35], providing a cohesive basis for the advantages observed with prebiotic enrichment in our analysis (origins of heterogeneity). Variability among studies likely indicates the following: (i) initial dietary fiber consumption and diet quality, which influence microbial substrates and short-chain fatty acid production; (ii) initial microbiome composition and host-microbe characteristics that affect engraftment and functionality; and (iii) product-specific factors (strain identity/viability, dosage, and delivery matrix). These factors likely underpin the ambiguous probiotic signal and the more reliable advantages of prebiotic-focused methodologies in our synthesis.

In clinical ramifications for adults with metabolic disorders, initial microbiome interventions should prioritize dietary fiber enhancement through prebiotic-rich foods or specific prebiotics, due to their molecular plausibility and clinical consistency [19-21,26,35]. Empiric probiotics may serve as adjuncts with clear strain/dose specifications and pragmatic expectations about personalized colonization [22,25]. Next-generation microorganisms, such as *Akkermansia*, are promising; however, they are still in the early stages of development. FMT for metabolic reasons should be regarded as experimental, with effects likely necessitating dietary and behavioral co-interventions [32,36]. Our trial-level patterns endorse these priorities.

Strengths and limitations of evidence that included trials were methodologically robust, but variations in outcome selection, diet reporting, strain integrity, and microbiome analytics hinder direct comparability and meta-analysis. Longer trials with objective goals (e.g., HbA1c, diabetes onset, NASH resolution), standardized microbiome/metabolite panels, and dietary adherence tracking are needed to determine who benefits and why [32-36]. 

Microbiome-directed therapy can reduce metabolic risk, but fiber-forward, prebiotic methods provide the most consistent benefits. Probiotics and FMT have context-dependent effects influenced by diet, baseline microbiota, and product features [19-22,26,32,35,36]. Personalisation and mechanistic targeting are likely to define the next generation of clinically relevant impact.

## Conclusions

Microbiome-targeted therapeutics can alleviate low-grade inflammation and cardiometabolic risk; however, the outcomes are variable. Fibre-forward, prebiotic approaches demonstrate the most consistent improvements in insulin resistance and central obesity, while probiotics exhibit effects that are reliant on individual response, and fecal microbiota transplantation remains supplementary and exploratory. A food-centric, individualized strategy, emphasizing dietary fiber and certain synbiotics, seems warranted, but forthcoming trials ought to standardize results and incorporate metabolomics to ascertain who derives the greatest benefit.
